# Female breast cancer in New South Wales, Australia, by country of birth: implications for health-service delivery

**DOI:** 10.1186/s12889-021-10375-x

**Published:** 2021-02-17

**Authors:** David Roder, George W. Zhao, Sheetal Challam, Alana Little, Elisabeth Elder, Gordana Kostadinovska, Lisa Woodland, David Currow

**Affiliations:** 1 Cancer Information and Analysis, Cancer Institute New South Wales, Level 4, 1 Reserve Road, St Leonards NSW 2065, PO Box 41, Alexandria, NSW 1435 Australia; 2 Equity, Multicultural Program, Cancer Institute New South Wales, St Leonards, NSW Australia; 3 Specialist Breast Surgery, Westmead Breast Cancer Institute, Westmead, NSW Australia; 4 Multicultural Health Service, Nepean Blue Mountains Local Health District, Penrith, NSW Australia; 5 Priority Populations, South Eastern Sydney Local Health District, Sydney, NSW Australia; 6 Cancer Institute New South Wales, St Leonards, NSW Australia

**Keywords:** Breast cancer survival, New South Wales, Birth countries

## Abstract

**Background:**

NSW has a multicultural population with increasing migration from South East Asia, the Western Pacific and Eastern Mediterranean.

**Objective:**

To compare cancer stage, treatment (first 12 months) and survival for 12 country of birth (COB) categories recorded on the population-based NSW Cancer Registry.

**Design:**

Historic cohort study of invasive breast cancers diagnosed in 2003–2016.

**Patients:**

Data for 48,909 women (18+ ages) analysed using linked cancer registry, hospital inpatient and Medicare and pharmaceutical benefits claims data.

**Measurement:**

Comparisons by COB using multivariate logistic regression and proportional hazards regression with follow-up of vital status to April 30th, 2020.

**Results:**

Compared with the Australia-born, women born in China, the Philippines, Vietnam and Lebanon were younger at diagnosis, whereas those from the United Kingdom, Germany, Italy and Greece were older. Women born in China, the Philippines, Vietnam, Greece and Italy lived in less advantaged areas. Adjusted analyses indicated that: (1) stage at diagnosis was less localised for women born in Germany, Greece, Italy and Lebanon; (2) a lower proportion reported comorbidity for those born in China, the Philippines and Vietnam; (3) surgery type varied, with mastectomy more likely for women born in China, the Philippines and Vietnam, and less likely for women born in Italy, Greece and Lebanon; (4) radiotherapy was more likely where breast conserving surgery was more common (Greece, Italy, and Lebanon) and the United Kingdom; and (5) systemic drug therapy was less common for women born in China and Germany. Five-year survival in NSW was high by international standards and increasing. Adjusted analyses indicate that, compared with the Australian born, survival from death from cancer at 5 years from diagnosis was higher for women born in China, the Philippines, Vietnam, Italy, the United Kingdom and Greece.

**Conclusions:**

There is diversity by COB of stage, treatment and survival. Reasons for survival differences may include cultural factors and healthier migrant populations with lower comorbidity, and potentially, less complete death recording in Australia if some women return to their birth countries for treatment and end-of-life care. More research is needed to explore the cultural and clinical factors that health services need to accommodate.

**Supplementary Information:**

The online version contains supplementary material available at 10.1186/s12889-021-10375-x.

## Background

Australia had a high relative survival from breast cancer at 93% 5 years from diagnosis in 2012–2016 [1, 2]. This is a marked increase from the corresponding 74% in 1982–87 [3] and was accompanied by a 37% decline at population level in age-standardized breast cancer mortality [4].

Survival from breast cancer in Australia, as indicated by the complement of the breast cancer mortality to incidence ratio (1-M/I), is at the high end of the international scale [5]. This varies across the population, however, with lower survival at each end of the age range and in women from lower socioeconomic areas [2, 6]. Screening participation through the “free” national BreastScreen program, which focuses on women aged 50–74 years, contributes to increases in post-diagnostic survival [7].

Effects on survival of differences in treatment access and treatment quality are not well documented although difficulties in access have been reported for older women and those from more disadvantaged areas [8]. While women from culturally and linguistically diverse (CALD) backgrounds have generally experienced a lower screening participation, differences in follow-up treatment access and quality for these women compared with the Australian-born are largely unknown [8, 9].

Australia has a multicultural population, reflected in New South Wales (NSW) where almost a third of the population live and where the percentage born outside of Australia has increased to about 28%, with 25% of residents speaking a language at home other than English [10, 11].

Between 2006 and 2016, the largest increases in population size from migration by birthplace applied to China, India, Nepal, the Philippines, Vietnam and South Korea [11, 12]. There was also an increase in people born in Lebanon [12].

Previous studies in NSW and other Australian jurisdictions indicated that compared with the Australian-born, migrants generally have a lower overall cancer incidence and mortality, although differences occur by cancer type and country of origin [13].

Few data exist in Australia on stage of breast cancer at diagnosis, or of treatment, by COB. Australia-wide cancer-registry studies have not monitored breast cancer treatment by COB or cultural background, although breast cancer treatment was investigated in an earlier regional study within the NSW capital, indicating that Asian women with node-positive, non-metastatic breast cancer were more likely to undergo mastectomy and to receive chemotherapy than non-Asian women [14]. No differences in exposure to radiotherapy or endocrine therapy were found in that study but the relevance of results to NSW overall was unknown [14]. Similarly, the relevance to NSW overall of data for Arabic-speaking women at a major Sydney hospital is not known [15].

The New South Wales Cancer Registry (NSWCR) records COB which shows population diversity [13]. We have used COB data in this study to investigate differences in stage (degree of spread), treatment and survival from breast cancer in NSW women according to whether born in Australia [9], other countries where numbers were sufficient for analysis, and for remaining women, according to whether born in a “mainly English-speaking” or “mainly non-English speaking” country.

The aim of the study was to analyse data for these women over a 14-year period using unadjusted and adjusted models by COB and other sociodemographic characteristics (age at diagnosis, socioeconomic status of residential area) and clinical features at diagnosis (stage and recorded comorbidity [16]). Differences in stage (degree of spread), treatment and survival from cancer were investigated.

## Methods

### Study design

A retrospective cohort design was used with linked NSWCR-recorded invasive breast cancers [16, 17], hospital inpatient [18], universal Medicare and pharmaceutical benefits claims data [19, 20], and death data from the NSW Register of Births, Deaths and Marriages and National Death Index [21]. The linkage process used nationally accepted and previously described privacy-protecting protocols to produce de-identified data for analysis [22]. Linkage of NSW-based datasets was performed by the Centre for Health Record Linkage whereas the Australian Institute of Health and Welfare (AIHW) linked nationally stored data [23]. Probabilistic data linkage was used plus best-practice data flows and procedures to protect privacy [24].

The study cohort comprised women aged 18+ years at diagnosis with invasive breast cancer (C50: ICD-10) diagnosed in 2003–2016, excluding multiple primary cancers [23]. Follow-up of cases continued to date of death or April 30th, 2020**,** whichever came first. Residents of four local health districts adjacent to NSW borders (10% of the NSW population) were excluded as many had care provided interstate, such that their treatments were not fully captured in NSW record systems. A sensitivity analysis of results by stage, treatments and cancer mortality outcomes according to exclusion or inclusion of these local health districts was undertaken, with similar results applying (Supplement Table 1).

Cohort members were classified by place of birth as: Australia; China; Germany; Greece; Italy; Lebanon; New Zealand; the Philippines; the United Kingdom; Vietnam; and for remaining members, “other mainly English speaking” or “mainly non-English speaking” countries respectively, as previously described [24, 25]. Members without a COB recorded on the NSWCR (6%) were excluded.

### Data collection and recording

The NSWCR uses international cancer registry standards to record primary cancer site, diagnosis date, demographic characteristics, stage (degree of spread), death date and whether death was caused by cancer, and residential address at diagnosis [17, 26]. Data sources comprise notifications from hospitals, pathology and related diagnostic sources, mandated through the NSW Public Health Act 2010 and the Cancer Institute (NSW) Act 2003 [17]. The National Death Index at the Australian Institute of Health and Welfare was used in this study to obtain death data, particularly for people diagnosed in NSW who died in another jurisdiction or in administrative districts of NSW not covered in this study [21]. The NSWCR is administered by Cancer Institute NSW, which is the NSW Government’s cancer control agency.

Consistent with conventional registry practice, clinical examination and test results for periods up to 4 months from diagnosis were used by the NSWCR to indicate primary cancer site and most advanced stage [26]. This was categorised as localised (confined to the site of origin), regional (had invaded adjacent tissues or regional nodes), or distant (had spread to distant lymph nodes or other organ sites), as defined in international guidelines [17, 24]. Cases with missing data on stage (4%) were classified as “unknown”. Place of residence was collected at census collection district level and coded using the Socio-Economic Index for Areas (SEIFA) which classifies areas by Index of Relative Socio-economic Disadvantage (IRSD) in quintiles [27].

Data on treatment by surgery, radiotherapy and/or systemic agents (chemotherapeutic, hormonal, immunological or targeted) were obtained for the 12 months following diagnosis, with surgery sub-classified as mastectomy or breast conserving. These data were obtained from linked NSW hospital data and Medicare and pharmaceutical benefits claims, both for procedures and pharmaceuticals, using the Australian Classification of Health Interventions (ACHI 8th edition) or corresponding Medicare and pharmaceutical benefits codes [18–20]. Hospital inpatient diagnostic codes (ICD-10) were also collected [18], with a 12-month look-back period, for deriving the Charlson Comorbidity Index [16, 28].

### Statistical analyses

Cross-tabulations were used in initial analyses to describe cohort characteristics by COB, age at diagnosis (broadly classified as 18–49, 50–59, 60–69, 70+ years), SEIFA quintile (1–5), diagnostic period (2003–04, 2005–06, 2007–08, 2009–10, 2011–12, 2013–14, 2015–16), stage (local, regional, distant, unknown), and recorded Charlson comorbidity index score [26] (0, ≥1) (Table 1). Characteristics for each COB were compared with those of the Australian-born, initially using the Mann-Whitney U Test for ordinal and Pearson Chi-square test for binary outcomes [29, 30]. These analyses tested the null hypothesis of no difference in the analysed characteristic with the Australian-born. The “*p* values” from statistical testing used in this context were unadjusted and not interpreted literally due to multiple testing. “*P* < 0.05” was used simply as a flag with “##” signifying potentially non-random differences.

**Table 1:** Sociodemographic and female breast cancer characteristics by country of birth in NSW; unadjusted comparisons with Australia*

	Australia	China mainland	Germ -any	Greece	Italy	Lebanon	New Zealand	Philipp-ines	United King- dom	Vietnam	Other mainly English-speaking	Other mainly non-English speaking
	% (n)	% (n)	% (n)	% (n)	% (n)	% (n)	% (n)	% (n)	% (n)	% (n)	% (n)	% (n)
**Age group (years)**	**Reference**	**##**	**##**	**##**	**##**	**##**	**##**	**##**	**##**	**##**	**##**	**##**
18 to 49	24.3 (7688)	39.6 (434)	11.4 (52)	6.5 (34)	8.6 (66)	29.9 (185)	34.9 (364)	34.5 (277)	19.2 (666)	44.8 (223)	30.4 (265)	28.3 (2000)
50 to 59	25.7 (8140)	27.2 (298)	23.8 (109)	19.2 (101)	16.0 (123)	33.6 (208)	31.0 (323)	38.0 (305)	23.1 (801)	30.7 (153)	26.7 (233)	27.2 (1919)
60 to 69	25.1 (7954)	17.0 (186)	32.3 (148)	34.5 (181)	30.3 (233)	24.2 (150)	19.8 (207)	20.4 (164)	27.8 (964)	17.9 (89)	26.7 (233)	24.4 (1722)
70+	25.0 (7909)	16.3 (179)	32.5 (149)	39.8 (209)	45.1 (347)	12.3 (76)	14.3 (149)	7.0 (56)	29.9 (1039)	6.6 (33)	16.3 (142)	20.1 (1423)
**SEIFA SES disadvantage**	**Reference**	**##**		**##**	**##**	**##**	**##**	**##**	**##**	**##**	**##**	**##**
1 (most)	17.0 (5401)	18.1 (199)	17.0 (78)	18.3 (96)	17.4 (134)	39.7 (246)	14.9 (155)	20.9 (168)	15.6 (540)	57.0 (284)	8.6 (75)	19.9 (1409)
2	18.5 (5853)	19.8 (217)	14.4 (66)	21.1 (111)	22.6 (174)	27.6 (171)	17.3 (180)	21.1 (169)	16.5 (573)	20.9 (104)	9.4 (82)	18.6 (1314)
3	18.9 (5974)	22.2 (244)	19.9 (91)	28.4 (149)	20.0 (154)	16.0 (99)	19.9 (208)	22.7 (182)	18.7 (650)	9.6 (48)	14.0 (122)	18.7 (1324)
4	21.7 (6868)	18.8 (206)	20.7 (95)	19.8 (104)	23.4 (180)	11.8 (73)	20.2 (211)	18.5 (148)	22.2 (770)	7.4 (37)	20.5 (179)	18.5 (1304)
5 (least)	24.0 (7595)	21.1 (231)	27.9 (128)	12.4 (65)	16.5 (127)	4.8 (30)	27.7 (289)	16.8 (135)	27.0 (937)	5.0 (25)	47.5 (415)	24.2 (1713)
**Year of diagnosis**	**Reference**	**##**	**##**			**##**		**##**	**##**	**##**		**##**
2003–2004	12.4 (3941)	9.4 (103)	17.2 (79)	13.3 (70)	12.9 (99)	9.5 (59)	11.4 (119)	11.2 (90)	12.9 (448)	10.2 (51)	11.8 (103)	10.6 (751)
2005–2006	12.8 (4053)	10.3 (113)	14.4 (66)	13.0 (68)	13.4 (103)	11.1 (69)	11.3 (118)	11.8 (95)	13.4 (464)	10.6 (53)	13.3 (116)	11.7 (825)
2007–2008	13.4 (4248)	11.6 (127)	11.4 (52)	11.0 (58)	15.2 (117)	11.3 (70)	13.9 (145)	10.6 (85)	14.5 (502)	12.0 (60)	13.4 (117)	13.4 (946)
2009–2010	14.3 (4528)	11.6 (127)	15.3 (70)	13.7 (72)	14.3 (110)	15.3 (95)	14.5 (151)	13.5 (108)	15.5 (537)	14.1 (70)	13.7 (120)	14.9 (1054)
2011–2012	14.6 (4628)	16.7 (183)	15.1 (69)	15.4 (81)	15.7 (121)	14.7 (91)	14.0 (146)	17.2 (138)	13.5 (470)	14.9 (74)	12.9 (113)	15.1 (1070)
2013–2014	15.5 (4902)	18.0 (198)	11.4 (52)	16.8 (88)	13.7 (105)	18.7 (116)	15.6 (163)	17.3 (139)	15.1 (524)	16.9 (84)	16.0 (140)	16.1 (1137)
2015–2016	17.0 (5391)	22.4 (246)	15.3 (70)	16.8 (88)	14.8 (114)	19.2 (119)	19.3 (201)	18.3 (147)	15.1 (525)	21.3 (106)	18.8 (164)	18.1 (1281)
**Stage (degree of spread)**	**Reference**			**##**	**##**	**##**						**##**
Localised	52.3 (16571)	51.1 (561)	47.6 (218)	47.8 (251)	46.7 (359	42.8 (265)	50.7 (529)	49.9 (400)	52.5 (1821)	53.2 (265)	51.0 (445)	49.0 (3464)
Regional	37.2 (11778)	39.8 (437)	40.2 (184)	38.9 (204)	41.5 (319)	45.7 (283)	39.6 (413)	40.1 (322)	36.4 (1264)	39.6 (197)	38.5 (336)	39.3 (2775)
Distant	6.3 (1995)	4.3 (47)	6.1 (28)	9.0 (47)	7.2 (55)	6.9 (43)	6..0 (63)	5.6 (45)	6.2 (214)	4.0 (20)	6.5 (57)	7.1 (504)
Unknown^a^	4.3 (1347)	4.7 (52)	6.1 (28)	4.4 (23)	4.7 (36)	4.5 (28)	3.6 (38)	4.4 (35)	4.9 (171)	3.2 (16)	4.0 (35)	4.5 (321)
**Comorbid Index (Charlson)**	**Reference**	**##**			**##**			**##**		^a^		**##**
0	96.8 (30688)	98.0 (1075)	95.6 (438)	95.8 (503)	94.7 (728)	97.4 (603)	98.6 (1017)	98.6 (791)	96.9 (3363)	^a^	97.6 (852)	97.5 (6889)
> 0	3.2 (1003)	2.0 (22)	4.4 (20)	4.2 (22)	5.3 (41)	2.6 (16)	1.4 (26)	1.4 (11)	3.1 (107)	^a^	2.4 (21)	2.5 (175)

Excludes multiple primary cancers and local health districts bordering other states/territories (see text)

*SEIFA SES* socio-economic indexes for areas socio-economic status

*##: *P* < 0.05 as calculated (see text)

^a^Excluded due to small numbers

Multiple logistic analyses were also undertaken of COB as a predictor (odds ratio - 95% confidence limits) for the following dependent variables: non-localised degree of spread (regional and distant), and in the 12 months from diagnosis, any recorded treatment, any surgery, mastectomy, breast conserving surgery, radiotherapy or systemic therapy respectively (i.e., 7 logistic models, adjusted for age, comorbidity status, SEIFA quintile, diagnostic year, and where relevant, stage at diagnosis (Table 2) [29, 30].

**Table 2:** Adjusted odds ratios for non-localised (regional and distant) stage of breast cancers at diagnosis by country of birth; New South Wales 2003–2016^a^

Predictor	Number (%) with non-localised stage (total 21,630, out of 46,779)	aOR non-localised (95% confidence limits)
**Country of birth**	Australia (ref.) *n* = 13,773 (45.4)	1.000
	China (mainland) *n* = 484 (46.3)	0.967 (0.853,1.095)
	**Germany** ***n*** **= 212 (49.3)**	**1.249 (1.031,1.512)**
	**Greece** ***n*** **= 251 (50.0)**	**1.301 (1.090,1.553)**
	**Italy** ***n*** **= 374 (51.0)**	**1.328 (1.146,1.539)**
	**Lebanon** ***n*** **= 326 (55.2)**	**1.400 (1.186,1.651)**
	New Zealand *n* = 476 (47.4)	1.038 (0.915,1.179)
	Philippines *n* = 367 (47.8)	1.050 (0.908,1.213)
	United Kingdom *n* = 1478 (44.8)	1.005 (0.935,1.081)
	Vietnam *n* = 217 (45.0)	0.862 (0.717,1.036)
	Other mainly English. Speaking *n* = 393 (46.9)	1.079 (0.939,1.239)
	**Other mainly non-English speaking** ***n*** **= 3279 (48.6)**	**1.123 (1.064,1.184)**
**Age at diagnosis (years)**	18 to 49 (ref.) *n* = 6348 (53.8)	1.000
	**50–59** ***n*** **= 5631 (45.6)**	**0.714 (0.678,0.751)**
	**60–69** ***n*** **= 4753 (40.0)**	**0.564 (0.535,0.594)**
	**70+** ***n*** **= 4898 (45.7)**	**0.699 (0.663,0.738)**
**SEIFA SES Disadvantage**	1 (most) (ref.) *n* = 4008 (48.0)	1.000
	2 *n* = 4057 (47.2)	0.960 (0.904,1.021)
	**3** ***n*** **= 4048 (45.8)**	**0.899 (0.847,0.956)**
	**4** ***n*** **= 4517 (46.3)**	**0.916 (0.863,0.973)**
	**5 (least)** ***n*** **= 5000 (44.5)**	**0.848 (0.801,0.899)**
**Year of diagnosis**	**Continuous (2003–16) n = 21,630**	**0.995 (0.991,1.000)**
**Comorbidity Index (Charlson)**	0 (ref.) *n* = 20,979 (46.1)	1.000
	**> 0** ***n*** **= 651 (50.3)**	**1.238 (1.106,1.385)**

^a^Female cases only – 95.6% with treatment details; Multivariate logistic regression adjusted for age, SES, diagnostic year and comorbidity; Excludes multiple primary cancers and local health districts bordering other states/territories (see text)

*aOR* adjusted odds ratio, *SEIFA SES* Socio-Economic Indexes for Areas, *ref* reference

Multivariable proportional hazards regression was undertaken to determine hazard ratios (95% confidence limits) for death from cancer up to 5 years from diagnosis by COB, adjusting for the same covariables as in the logistic analyses and for treatment by surgery, radiotherapy and systemic therapy (Table 2). Follow-up of survival was from diagnosis to April 30th, 2020, or death, whichever came first [29, 30].

All multivariate analyses were complete-case analyses excluding the 4% with unknown stage.

## Results

### Descriptive characteristics by country of birth (unadjusted)

Compared with Australian women as the reference category, the following differences were found that were potentially non-random, i.e., flagged as “##”, as described in the Methods (see Table 1):

- A younger age distribution at diagnosis presented for women born in China, Lebanon, New Zealand, the Philippines, Vietnam, and other “mainly English speaking” and “mainly non-English speaking” countries of birth; and an older age distribution for women born in Germany, Greece, Italy, and the United Kingdom.
- A greater socioeconomic disadvantage was evident for women born in China, Greece, Italy, Lebanon, the Philippines, Vietnam, and “other mainly non-English speaking” countries; and less disadvantage for women born in New Zealand, the United Kingdom, and “other mainly English-speaking” countries.
- Less recent diagnostic years applied for women born in Germany and the United Kingdom; and more recent diagnostic years for women born in China, Lebanon, the Philippines, Vietnam, and “other mainly non-English speaking” countries.
- More localised stage was not observed for any country category, but less localised stage was evident for women born in Greece, Italy, Lebanon, and “other mainly non-English speaking” countries.
- More frequent recording of comorbidity applied for women born in Italy and less frequent recording of comorbidity for women born in China, the Philippines, and “other mainly non-English speaking” countries. Women born in Vietnam also recorded comorbidity less frequently, but the number of women affected is not presented due to small cell size (*n* < 5).

### Country of birth as a predictor of stage (adjusted)

Multiple logistic analyses indicated an elevated adjusted odds ratio (aOR) of non-localised (regional and distant) stage for women born in Germany, Greece, Italy, Lebanon and “other mainly non-English speaking” countries (Table 2). Recorded comorbidity was also associated with an elevated aOR for non-localised stage whereas a lower aOR was associated with lower residential disadvantage (higher SES), older age of 50+ years, and although the difference was small (approximate 6% across the study period), potentially with a more recent diagnostic year at aOR 0.995 (0.991–1.000).

Results from supplementary analysis comparing COB with Australia were similar, irrespective of inclusion or exclusion of local health districts adjacent to NSW borders (Supplement Table 1).

### Country of birth as a predictor of treatment (first 12 months)

#### Any treatment (surgery, radiotherapy, systemic therapy)

Unadjusted analysis showed most women born in Australia (98.5%) were recorded as having treatment, as was the case for women born in Lebanon. The percentage having any treatment had a narrow range from 97.6% (“other mainly non-English speaking” countries) to 98.9% (the Philippines). When adjusted for sociodemographic and clinical variables, the aOR (95% confidence limits) differed from the Australian-born as the reference category for three country categories with a lower aOR for women born in China, New Zealand, and “other mainly English-speaking” countries. Other differences included a low aOR for any treatment for distant compared with localised spread and presence of recorded comorbidity. Women living in the highest socioeconomic (least disadvantaged) areas had a higher aOR for any treatment compared with women from the most disadvantaged areas. A lower likelihood of any treatment was also indicated with older age.

Supplementary analysis comparing results by COB showed similar results irrespective of inclusion or exclusion of local health districts adjacent to NSW borders (Supplement Table 1).

#### Any surgery

Surgery was the most common treatment, applying to 91.6% of Australian-born women and ranging by COB category from 87.6% (Greece) to 95.2% (Vietnam). After adjustment for other sociodemographic and clinical variables, only women born in “mainly non-English speaking” countries had a different aOR for surgery that was lower than for the Australian-born (Table 3). Lower aORs also applied for older age at diagnosis, more recent diagnostic year, regional and distant compared with localised stage at diagnosis, and presence of recorded comorbidity (Table 3). Compared with the most disadvantaged quintile, women living in less disadvantaged areas (quintiles 2–5) had elevated odds ratios, peaking at 1.484 (1.296, 1.700) for the least disadvantaged (highest SES) quintile.

**Table 3:** Adjusted odds ratios for treatment of female breast cancers (1st 12 months from diagnosis) by country of birth; New South Wales 2003–2016^a^

Predictor	Total number cases (*n* = 46,779)	aOR Any treatment (*n* = 46,297)	aOR Surgery (*n* = 43,653)	aOR Mastectomy (*n* = 18,283)	aOR Conserving surgery (n = 25,327)	aOR Radiotherapy (*n* = 31,132)	aOR Systemic therapy (n = 39,231)
**Country of birth**	Australia (*n* = 30,344)	1.000	1.000	1.000	1.000	1.000	1.000
	China (mainland) (*n* = 1045)	**0.463 (0.257,0,835)**	1.021 (0.744,1.401)	**1.625 (1.430,1.846)**	**0.605 (0.531,0.689)**	**0.610 (0.535,0.695)**	**0.715 (0.605,0.845)**
	Germany (*n* = 430)	0.795 (0.315,2.004)	0.835 (0.554,1.258)	0.838 (0.683,1.028)	1.146 (0.935,1.405)	1.134 (0.916,1.404)	**0.683 (0.535,0.871)**
	Greece (*n* = 502)	0.885 (0.398,1.965)	0.792 (0.553,1.132)	**0.757 (0.624,0.917)**	**1.254 (1.073,1.516)**	**1.313 (1.076,1.603)**	0.984 (0.768,1.260)
	Italy (*n* = 733)	2.279 (0.897,5.793)	1.059 (0.766,1.465)	**0.734 (0.626,0.861)**	**1.403 (1.198,1.644)**	**1.431 (1.210,1.692)**	1.153 (0.933,1.425)
	Lebanon (*n* = 591)	2.399 (0.579,9.935)	1.116 (0.750,1.660)	**0.614 (0.512,0.737)**	**1.666 (1.393,1.994)**	**1.518 (1.242,1.857)**	1.034 (0.802,1.333)
	New Zealand (*n* = 1005)	**0.484 (0.262,0.892)**	0.854 (0.634,1.151)	1.066 (0.934,1.217)	0.918 (0.804,1.048)	0.938 (0.815,1.080)	0.961 (0.798,1.156)
	Philippines (*n* = 767)	1.026 (0.373,2.824)	1.254 (0.846,1.858)	**1.477 (1.273,1.714)**	**0.693 (0.596,0.807)**	**0.646 (0.555,0.752)**	1.009 (0.811,1.256)
	United Kingdom (*n* = 3299)	0.917 (0.633,1.328)	0.929 (0.787,1.097)	**0.911 (0.844,0.984)**	**1.080 (1.001,1.167)**	**1.130 (1.042,1.226)**	0.986 (0.892,1.091)
	Vietnam (*n* = 482)	1.327 (0.315,5.593)	1.178 (0.709,1.955)	**1.542 (1.278,1.859)**	**0.643 (0.532,0.778)**	**0.646 (0.534,0.781)**	0.903 (0.695,1.174)
	Other mainly English speaking (*n* = 838)	0.827 (0.370,1.851)	1.125 (0.789,1.605)	1.130 (0.978,1.305)	0.893 (0.772,1.033)	0.948 (0.812,1.107)	0.845 (0.696,1.027)
	Other mainly non-English speaking (*n* = 6743)	**0.491 (0.385,0.627)**	**0.750 (0.667,0.843)**	0.958 (0.906,1.014)	0.984 (0.930,1.041)	0.953 (0.899,1.011)	**0.801 (0.745,0.863)**
**Age at diagnosis (18 + years)**	Continuous (n = 46,779)	**0.913 (0.903,0.923)**	**0.932 (0.926,0.937)**	**1.013 (1.010,1.017)**	**0.965 (0.962,0.968)**	**0.911 (0.908,0.915)**	**0.942 (0.938,0.945)**
**SEIFA SES Disadvantage**	1 (most) (ref.) (*n* = 8346)	1.000	1.000	1.000	1.000	1.000	1.000
	2 (*n* = 8591)	0.983 (0.754,1.281)	**1.157 (1.016,1.317)**	0.962 (0.903,1.026)	**1.080 (1.012,1.152)**	**1.081 (1.012,1.155)**	1.007 (0.926,1.095)
	3 (*n* = 8847)	1.229 (0.919,1.645)	**1.249 (1.094,1.427)**	0.967 (0.908,1.031)	**1.096 (1.028,1.168)**	**1.121 (1.049,1.198)**	1.050 (0.965,1.141)
	4 (*n* = 9751)	**1.370 (1.018, 1.844)**	**1.176 (1.032,1.340)**	**0.926 (0.870,0.985)**	**1.127 (1.058,1.200)**	**1.222 (1.145,1.305)**	**1.132 (1.041,1.230)**
	5 (least) (*n* = 11,244)	**2.068 (1.480,2.889)**	**1.484 (1.296,1.700)**	0.928 (0.891,1.012)	**1.158 (1.090,1.232)**	**1.309 (1.228,1.396)**	**1.153 (1.063,1.251)**
**Year of diagnosis**	Continuous (2003 to 2016) (n = 46,779)	**0.995 (0.991,1.000)**	**0.982 (0.971,0.992)**	**0.981 (0.976,0.985)**	**1.015 (1.010,1.020)**	**0.644 (0.595,0.698)**	**1.054 (1.047,1.061)**
**Stage (degree of spread)**	Local (ref.) (n = 25,149)	1.000	1.000	1.000	1.000	1.000	1.000
	Regional (n = 18,512)	0.806 (0.610,1.065)	**0.732 (0.660,0.813)**	**2.809 (2.699,2.923)**	**0.342 (0.329,0.356)**	**1.355 (1.298,1.414)**	**4.277 (4.007,4.565)**
	Distant (*n* = 3118)	**0.058 (0.046,0.073)**	**0.030 (0.027,0.033)**	1.010 (0.931,1.097)	**0.114 (0.103,0.125)**	**0.644 (0.595,0.698)**	**2.356 (2.111,2.631)**
**Comorbidity Index (Charlson)**	0 (ref.) (n = 45,486)	1.000	1.000	1.000	1.000	1.000	1.000
	> 0 (*n* = 1293)	**0.246 (0.191,0.317)**	**0.314 (0.266,0.371)**	**0.874 (0.774,0.986)**	**0.632 (0.558,0.717)**	**0.472 (0.416,0.535)**	**0.680 (0.595,0.778)**

^a^(95% confidence limits); adjusted for age at diagnosis, SES, year of diagnosis, stage and comorbidity index; Multivariate logistic regression (see text) Excludes multiple primary cancers and local health districts bordering other states/territories (see text). Treatment data for women with a recorded stage; numbers receiving specified treatment in brackets under column headings

*aOR* adjusted odds ratio, *SEIFA SES* socio-economic indexes for areas, *ref* reference

Supplementary analysis comparing results by COB showed little differences irrespective of inclusion or exclusion of local health districts adjacent to NSW borders (Supplement Table 1).

#### Mastectomy

Of Australian-born women, 38.3% had a mastectomy, with the percentage ranging from 30.7% (women born in Lebanon) to 49.0% (women born in China and Vietnam). Compared with Australian-born, and after adjusting for sociodemographic and clinical variables, the aOR for having a mastectomy was lower for women born in Greece, Italy, Lebanon and the United Kingdom, but higher for women born in China at 1.625 (1.430, 1.846), the Philippines at 1.477 (1.273, 1.714), and Vietnam at 1.542 (1.278, 1.859) (Table 3). Other variables associated with mastectomy after adjustment (aOR) included lower odds for more recent diagnostic years and recorded comorbidity, but a higher aOR for older age and regional compared with localised stage.

#### Breast conserving surgery (BCS)

The proportion of Australian-born women having BCS was 53.2%, varying from 43.0% (China) to 61.1% (Lebanon). After adjusting for other sociodemographic and clinical variables, the aOR for having BCS was lower than for the Australian-born for women born in China at 0.605 (0.531, 0.689), the Philippines at 0.693 (0.596, 0.807), and Vietnam at 0.643 (0.532, 0.778); and higher than for the Australian-born for those born in Greece at 1.254 (1.073, 1.516), Italy at 1.403 (1.198, 1.644), Lebanon at 1.666 (1.393, 1.994), and the United Kingdom at 1.080 (1.001, 1.167) (Table 3). A lower aOR also applied for older age at diagnosis, recorded comorbidity, and compared with localised stage at diagnosis, regional and distant stage, and a higher aOR for more recent diagnostic years. Compared with the most disadvantaged quintile, women living in the less disadvantaged areas (quintiles 2–5) had elevated aORs, for BCS peaking at 1.158 (1.090, 1.232) for the least disadvantaged (highest SES) quintile 5.

Supplementary analysis comparing results by COB showed little differences irrespective of inclusion or exclusion of local health districts adjacent to NSW borders (Supplement Table 1).

#### Radiotherapy

The proportion of women having radiotherapy varied with surgery type from 39.2% for those having a mastectomy to 89.1% for those having BCS. Overall, the proportion of women having radiotherapy was 68.2% for all women in aggregate. This varied by COB with 65.0% for the Australian-born and ranging from 58.0% for China to 76.1% for women born in Lebanon. Compared with the Australian-born, a lower aOR for radiotherapy applied to women born in China at 0.610 (0.535, 0.695), the Philippines at 0.646 (0.555, 0.752), and Vietnam at 0.646 (0.534, 0.781); whereas a higher aOR applied to women born in Greece at 1.313 (1.076, 1.603), Italy at 1.431 (1.210, 1.692), Lebanon at 1.518 (1.242, 1.857) and the United Kingdom at 1.130 (1.042, 1.226). Older women were less likely to have radiotherapy while higher use of radiotherapy was indicated for regional compared with local spread of disease and less disadvantaged quintiles 2–5 than quintile 1, peaking at 1.309 (1.228, 1.396) for quintile 5 (least disadvantaged). Cancers with distant spread were less likely to have radiotherapy than localised cancers. Comorbidity was also associated with a lower likelihood of radiotherapy (Table 3).

Supplementary analysis comparing results by COB showed little differences irrespective of inclusion or exclusion of local health districts adjacent to NSW borders (Supplement Table 1).

#### Systemic therapy

The proportion of women having systemic therapy tended to be higher for women having a mastectomy (87.7%) than BCS (81.6%). Overall, the proportion of women having systemic therapy was 84.2% for all women in aggregate. This varied by COB with 83.7% of Australian-born having systemic therapy and with a range from 77.3% (Germany) to 86.3% (Lebanon). Compared with the Australian born, the aOR for systemic therapy was lower for women born in China at 0.715 (0.605, 0.845), Germany at 0.683 (0.535, 0.871), and “other mainly non-English speaking” countries at 0.801 (0.745, 0.863). The aOR for systemic therapy was also lower at older ages and where comorbidity was recorded, but higher for upper socioeconomic quintiles 4 and 5 compared with the lowest quintile 1, and for more recent diagnostic years. Compared with localised stage at diagnosis, the aOR for systemic therapy was higher for regional stage at 4.277 (4.007, 4.565) and distant stage at 2.356 (2.111, 2.631).

Supplementary analysis comparing results by COB showed little differences irrespective of inclusion or exclusion of local health districts adjacent to NSW borders (Supplement Table 1).

### Country of birth as a predictor of cancer death

The five-year survival from death Australian-born patients was 84.1%, varying from 79.8% (Italy) to 93.6% (Vietnam). Compared with the Australian-born, the adjusted risk of cancer death (hazard ratio) within 5 years of diagnosis was lower for women born in China at 0.597 (0.467,0.762), Greece at 0.634 (0.491,0.82), Italy at 0.795 (0.652,0.97), the Philippines at 0.698 (0.54,0.902), the United Kingdom at 0.880 (0.783,0.991), Vietnam at 0.469 (0.282,0.78), and “other mainly non-English speaking” countries at 0.787 (0.72,0.859) (Table 4). Other predictors of a lower risk of cancer death (hazard ratio) after adjusting for sociodemographic and clinical characteristics were: (a) compared with the lowest socioeconomic residential area (quintile 1), for higher socioeconomic quintiles 3–5 with the lowest adjusted hazards ratio applying for the highest quintile 5 at 0.684 (0.624,0.75), and for more recent diagnostic years. By comparison, other predictors of higher adjusted hazard ratio (aHR) included: older age at diagnosis, recorded comorbidity, and compared with localised cancer, regional stage at 2.921 (2.739,3.115) and distant stage at 7.115 (6.485,7.807). Receiving surgical treatment was associated with a lower aHR of 0.285 (0.263,0.308), compared with 0.768 (0.724,0.814) for radiotherapy and 0.649 (0.60,0.702) for systemic therapy.

**Table 4:** Adjusted hazard ratios for death from cancer following female breast cancer diagnosis by country of birth; New South Wales 2003–2016^a^

Predictor	Number of cancer deaths (%) for follow-up period (total 6023 deaths out of 46,779 cases)	aHR death from cancer (95% confidence limits)
Country of birth	Australia (ref.) *n* = 4129 (13.6)	1.000
	**China (mainland) n = 73 (7.0)**	**0.597 (0.467,0.762)**
	Germany *n* = 70 (16.3)	0.957 (0.743,1.232)
	**Greece** ***n*** **= 66 (13.1)**	**0.634 (0.491,0.820)**
	**Italy** ***n*** **= 125 (17.1)**	**0.795 (0.652,0.970)**
	Lebanon *n* = 71 (12.0)	0.869 (0.681,1.108)
	New Zealand *n* = 101 (10.0)	0.922 (0.745,1.140)
	**Philippines** ***n*** **= 54 (7.0)**	**0.698 (0.540,0.902)**
	**United King.** ***n*** **= 460 (13.9)**	**0.880 (0.783,0.991)**
	**Vietnam n = 25 (5.2)**	**0.469 (0.282,0.780)**
	Other mainly English- speaking *n* = 87 (10.4)	1.013 (0.810,1.268)
	**Other mainly non-English speaking** ***n*** **= 762 (11.3)**	**0.787 (0.720,0.859)**
Age at diagnosis (18 to 80+ years)	**Continuous** ***n*** **= 6023 (12.9)**	**1.066 (1.063,1.070)**
SEIFA SES Disadvantage	1 (most) (ref.) *n* = 1419 (17.0)	1.000
	2 *n* = 1335 (15.5)	0.946 (0.866,1.034)
	**3** ***n*** **= 1158 (13.1)**	**0.895 (0.820,0.977)**
	**4** ***n*** **= 1143 (11.7)**	**0.835 (0.764,0.912)**
	**5 (least)** ***n*** **= 968 (8.6)**	**0.684 (0.624,0.750)**
Year of diagnosis (2003 to 2016)	**Continuous (2013–16) n = 6023 (12.9)**	**0.964 (0.957,0.972)**
Stage (degree of spread)	**Local (ref.)** ***n*** **= 1565 (6.2)**	**1.000**
	**Regional** ***n*** **= 2747 (14.8)**	**2.921 (2.739,3.115)**
	**Distant** ***n*** **= 1711 (54.9)**	**7.115 (6.485,7.807)**
Comorbidity Index (Charlson)	**0 (ref.)** ***n*** **= 5392 (11.9)**	**1.000**
	**> 0** ***n*** **= 631 (48.8)**	**1.958 (1.727,2.219)**
Treatment received (1st round)	**Surgery** ***n*** **= 4167 (9.5)**	**0.285 (0.263,0.308)**
	**Radiotherapy** ***n*** **= 2779 (8.9)**	**0.768 (0.724,0.814)**
	**Systemic** ***n*** **= 4778 (12.2)**	**0.649 (0.60,0.702)**

^a^Adjusted for age at diagnosis, SES, year of diagnosis, stage, comorbidity and treatment; Multivariate proportional hazard regression (see text) Excludes multiple primary cancers and local health districts bordering other states/territories. Date of censoring of live cases 5 years postdiagnosis or April 30th 2020, whichever came first (see text)

*aHR* adjusted hazards ratio, *SEIFA SES* socio-economic indexes for areas socio-economic status, *ref* reference

Supplementary analysis comparing results by COB showed little differences irrespective of inclusion or exclusion of local health districts adjacent to NSW borders (Supplement Table 1).

## Discussion

Sociodemographic trends for women in NSW diagnosed with breast cancer, as presented in this study, have paralleled Australian immigration patterns with increasing numbers of migrants from China, the Philippines, Vietnam, and Lebanon [10, 11]. There were higher percentages of women from European birth countries such as Germany and the United Kingdom in the earlier diagnostic years, and higher percentages from China, the Philippines, Vietnam and Lebanon in the more recent years. A difference by age at diagnosis was also evident, with patients from Germany, Greece, Italy, and the United Kingdom tending to be older and those from China, the Philippines, Vietnam and Lebanon tending to be younger. This pattern reflects the migration composition to Australia with large numbers of migrants from Europe after World War II [10, 11].

Socioeconomic disadvantage appeared to be more pronounced for patients born outside of Australia, including in China, the Philippines, Vietnam and Lebanon, and for those born in Italy and Greece. These patterns have implications for service provision if services are to meet the cultural and language requirements of NSW breast cancer patients. A positive feature of those born outside of Australia (e.g., in China, the Philippines, Vietnam, and “other mainly non-English speaking” countries) was less evidence of comorbidity. While this may reflect variations in reporting due to differences in culture, health beliefs and practices, it may also reflect health requirements at time of migration, leading to selection of the more resilient who could better withstand comprehensive cancer treatments and associated toxicities [31].

Predictably non-localised stage at diagnosis was associated with increased risk of cancer death in the five years following diagnosis. Reasons for the lower adjusted risk of non-localised disease in the least disadvantaged (upper SES) areas require investigation, including the possible roles of higher health literacy and more active health-seeking behaviour. A need for more active promotion of early-detection in women from the more disadvantaged areas, and women born in Greece, Italy, Lebanon, and “other mainly non-English-speaking” countries, is apparent from this study. Many of the older women from these countries migrated after World War II and are more likely to have lower levels of education, and lower health literacy. They also may have less familiarity with the health system and roles of health professionals, and when seeking services, face additional financial, cultural, language and social barriers. Barriers may be increased if older women revert with increasing age to their original language. BreastScreen records should be examined to assess the contribution of lower screening coverage to elevated numbers of women with non-localised cancers by COB [2].

Meanwhile the lower proportion of non-localised breast cancer stage from age 50 years may reflect the cumulative effects on stage of population screening, with active recruitment from age 50 years [7, 32]. Also, premenopausal women can have more aggressive breast cancers that present at a later symptomatic stage, which could have affected these age differences.

Mastectomies were more common in women from China, the Philippines and Vietnam, whereas breast conserving surgery was more common in women from Greece, Italy and Lebanon. Again, cultural factors may have played a part in these differences, including in some instances, a lower trust in the health system and stronger reliance on collective decision-making within families. Our hypothesis is that differences in breast size also may have contributed, but substantiating international data are lacking [33].

Older women were more likely to have a mastectomy and less likely to have breast conserving surgery. Again, this may be a cultural effect or reflect a lower interest in breast retention for cosmetic reasons in older age. Also, some older women, particularly those from country regions, may prefer to have a mastectomy to avoid the need to travel to urban centres for adjuvant radiotherapy, as recommended for most women following breast conserving surgery [34]. The finding that women from the most disadvantaged residential areas were less likely to have breast conserving surgery is consistent with earlier research evidence [34]. Anecdotally some women reportedly prefer mastectomy because they believe it is more likely to clear the cancer [34]. Predictably mastectomy was less common for cancers of localised and distant stage, whereas breast conserving surgery was less common for regional and distant stage. Meanwhile recorded comorbidity was associated with a lower likelihood of surgery, both mastectomy and breast conserving surgery.

While exposures to radiotherapy and systemic therapy were analysed as discrete entities to indicate overall levels of exposure by country of birth, sub-analyses predictably indicated exposure to radiotherapy to be higher in BCS cases (89%) than those having a mastectomy (39%). The difference was less marked for systemic therapy (88% exposure for mastectomy v. 82% for BCS).

Overall, radiotherapy was less likely for women from China, the Philippines and Vietnam which accompanied a lower exposure to breast conserving surgery. By comparison women from Greece, Italy, the United Kingdom and Lebanon were more likely to have radiotherapy. Likelihood of radiotherapy was also lower in older women and those with recorded comorbidity, which may reflect an increased frailty and lack of resilience to travel to radiotherapy centres and to withstand treatment. The potential to further increase support networks to access radiotherapy services should be considered, including among the socially isolated, older people who lack carers, and multicultural people who do not speak English and whose family networks live outside of Australia.

Women from the least disadvantaged areas were more likely to have radiotherapy which is consistent with their higher use of breast conserving surgery. Differing health beliefs may also impact on uptake of radiotherapy and systemic therapy.

Overall, systemic therapies were less likely to be used by women born in China and Germany, older women, and those with recorded comorbidity. Women from more disadvantaged areas tended to have less exposure to systemic therapy. The reasons require further study. Predictably, systemic therapy was more commonly used for non-localised stage.

Survival from cancer was lower in older age, those reporting more comorbidity, and more non-localised stage, and higher in the least disadvantaged, which accords with previous study results [2, 6]. The association of lower survival with treatment, particularly surgery, is plausible.

The higher survival for women who migrated to Australia from China, the Philippines, Vietnam and “other mainly non-English speaking” countries was unexpected. Several possible explanations exist. As an observational study using routinely collected data, we suspect that residual confounding may have occurred from a range of sociodemographic and clinical factors due to insufficient measurement precision or from cultural and other health-related confounders for which we had no data. Other possible explanations include health requirements for acceptance of migrants to Australia which may predispose to acceptance of those with greater health resilience [31]. Also missing death data may have contributed if some migrants returned to their birth countries in the terminal stages such that their deaths were not recorded in Australia. Further research with customized data is needed to explore these possibilities, as indicated in the Discussion.

Regarding loss of 10% of cases by excluding border local health districts with potential for interstate treatment, sensitivity analysis indicated that this had little effect on results. Of 77 comparisons of effects by country of birth (Supplement Table 1), 70 gave similar results based on 95% confidence limits, and the few differences found were small, indicating reliability of results.

This study has benefited from access to data from a high-quality population-based registry extended through data linkage to include hospital inpatient and health insurance data to indicate clinical management and indicators of comorbidity.

Limitations include incompleteness of data on services provided in non-admitted outpatient settings in public hospitals. We consider that key surgical and radiotherapy data would have been captured, plus systemic therapy data for care provided in inpatient settings and in private hospital outpatient and out-of-hospital environments, which are generally covered by universal Medicare benefits insurance for both procedures and pharmaceuticals. Another limitation was exclusion of 10% of breast cancers due to place of residence being in local health districts adjacent to the NSW border where potential existed for interstate public hospital treatment that would not have been recorded in NSW data systems. Lack of data on language spoken at home or requests for interpreter services was also a limitation as these features can be strong markers of cultural diversity.

Often odds ratios and hazard ratios have been reported as indicators of potential non-random differences when divergences from Australian-born as the reference category were small. Some divergences were small because small units of measurement were used (e.g., single years for age and calendar year). In other instances, the effect of COB on outcomes could have been diluted substantially depending on time of arrival in Australia (e.g., whether during childhood or in adulthood closer to the breast-cancer diagnosis date). For this reason, all potentially non-random differences, however small, have been reported and interpreted as flagging differences of potential importance for consideration and further investigation.

## Conclusions

These data indicate cultural diversity in stage, type of surgical treatment, exposure to radiotherapy and systemic drug therapy, and survival from cancer. Reasons may include cultural factors and migration health requirements. This and differences in reporting may explain lower reported comorbidity levels, and potentially less complete death recording in Australia if some women born outside of Australia were to return to their birth countries for treatment and end-of-life care and die there. More research is needed to investigate these and other possible explanations. Meanwhile, results illustrate the sociodemographic, cultural and clinical diversity of women with breast cancer in NSW and associations with prognosis, clinical care and outcomes by COB. This diversity presents challenges that NSW health services are addressing and need to be accommodated. At present, survival from breast cancer in NSW is at the high end of the international scale and increasing.

## Supplementary Information

**Additional file 1: Table S1.** Adjusted odds ratios (95% CIs) for non-localised (regional and distant) stage and specified treatments, and hazard ratios (95% CIs) for death from cancer, by country of birth compared with Australia as the reference category. Analyses first excluding and then including local health districts (LHDs) adjacent to the New South Wales border; Female breast cancers 2003–2016.

## Data Availability

Data are available from the authors upon reasonable request and with permission of the NSW Cancer Institute and Australian Institute of Health and Welfare.

## Abbreviations

COB: Country of birth
CALD: Culturally and linguistically diverse
NSWCR: New South Wales Cancer Registry
AIHW: Australian Institute of Health and Welfare
SEIFA: Socio-economic indexes for areas
IRSD: Index of Relative Socio-economic Disadvantage (IRSD)
ACHI: Australian Classification of Health Interventions (8th edition)
aOR: Adjusted odds ratio
SES: Socio-economic status
BCS: Breast conserving surgery
AHR: Adjusted hazards ratio
