# Supplementary material for: Female breast cancer in New South Wales, Australia, by country of birth: implications for health-service delivery
Source: BMC Public Health. 2021 Feb 17;21:371. doi: 10.1186/s12889-021-10375-x (PMC7890625; doi:10.1186/s12889-021-10375-x)
Supplement: Supplementary file 1 — Additional file 1: Table S1. Adjusted odds ratios (95% CIs) for non-localised (regional and distant) stage and specified treatments, and hazard ratios (95% CIs) for death from cancer, by country of birth compared with Australia as the reference category. Analyses first excluding and then including local health districts (LHDs) adjacent to the New South Wales border; Female breast cancers 2003–2016. [file 12889_2021_10375_MOESM1_ESM.docx]

**Supplement Table 1:** Adjusted odds ratios (95% CIs) for non-localised (regional and distant) stage and specified treatments, and hazard ratios (95% CIs) for death from cancer, by country of birth compared with Australia as the reference category. Analyses first excluding and then including local health districts (LHDs) adjacent to the New South Wales border; Female breast cancers 2003-2016

|  |  | **Adjusted odds ratios for specified outcomes*** | | | | | | **Adjusted hazard ratios**** |
| --- | --- | --- | --- | --- | --- | --- | --- | --- |
| **Country of birth** | **Including border LHDs** | **Regional and**  **distant vs local stage** | **Any treatment** | **Mastectomy** | **Breast conserving surgery** | **Radiotherapy** | **Systemic**  **therapies** | **Death from**  **cancer** |
| **Australia (reference)** | No | 1.000 | 1.000 | 1.000 | 1.000 | 1.000 | 1.000 | 1.000 |
|  | Yes | 1.000 | 1.000 | 1.000 | 1.000 | 1.000 | 1.000 | 1.000 |
| **China (mainland)** | No | 0.967  (0.853,1.095) | **0.463**  **(0.257,0.835)** | **1.625**  **(1.430,1.846)** | **0.605**  **(0.531,0.689)** | **0.610**  **(0.535,0.695)** | **0.715**  **(0.605,0.845)** | **0.597**  **(0.467,0.762)** |
|  | Yes | 0.967  (0.854,1.095) | **0.551**  **(0.308,0.986)** | **1.680**  **(1.480,1.907)** | **0.643**  **(0.565,0.732)** | **0.609**  **(0.535,0.694)** | **0.694**  **(0.587,0.819)** | **0.598**  **(0.469,0.763)** |
| **Germany** | No | **1.249**  **(1.031,1.512)** | 0.795  (0.315,2.004) | 0.838  (0.683,1.028) | 1.146  (0.935,1.405) | 1.134  (0.916,1.404) | **0.683**  **(0.535,0.871)** | 0.957  (0.743,1.232) |
|  | Yes | **1.250**  **(1.040,1.501)** | 0.566  (0.270,1.185) | 0.849  (0.698,1.032) | 1.148  (0.945,1.393) | 1.114  (0.908,1.365) | **0.672**  **(0.532,0.850)** | 0.990  (0.779,1.258) |
| **Greece** | No | **1.301**  **(1.090,1.553)** | 0.885  (0.398,1.965) | **0.757**  **(0.624,0.917)** | **1.254**  **(1.073,1.516)** | **1.313**  **(1.076,1.603)** | 0.984  (0.768,1.260) | **0.634**  **(0.491,0.820)** |
|  | Yes | **1.291**  **(1.083,1.539)** | 1.050  (0.476,2.317) | **0.787**  **(0.651,0.952)** | **1.296**  **(1.074,1.563)** | **1.288**  **(1.057,1.568)** | 0.945  (0.741,1.207) | **0.669**  **(0.523,0.856)** |
| **Italy** | No | **1.328**  **(1.146,1.539)** | 2.279  (0.897,5.793) | **0.734**  **(0.626,0.861)** | **1.403**  **(1.198,1.644)** | **1.431**  **(1.210,1.692)** | 1.153  (0.933,1.425) | **0.795**  **(0.652,0.970)** |
|  | Yes | **1.308**  **(1.134,1.507)** | **2.381**  **(1.018,5.562)** | **0.764**  **(0.655,0.890)** | **1.440**  **(1.238,1.676)** | **1.432**  **(1.219,1.683)** | 1.106  (0.904,1.353) | 0.785  (0.642,0.960) |
| **Lebanon** | No | **1.400**  **(1.186,1.651)** | 2.399  (0.579,9.935) | **0.614**  **(0.512,0.737)** | **1.666**  **(1.393,1.994)** | **1.518**  **(1.242,1.857)** | 1.034  (0.802,1.333) | 0.869  (0.681,1.108) |
|  | Yes | **1.397**  **(1.185,1.648)** | 2.704  (0.656,11.137) | **0.632**  **(0.527,0.759)** | **1.783**  **(1.491,2.131)** | **1.525**  **(1.248,1.864)** | 1.002  (0.778,1.292) | 0.887  (0.698,1.128) |
| **New Zealand** | No | 1.038  (0.915,1.179) | **0.484**  **(0.262,0.892)** | 1.066  (0.934,1.217) | 0.918  (0.804,1.048) | 0.938  (0.815,1.080) | 0.961  (0.798,1.156) | 0.922  (0.745,1.140) |
|  | Yes | 1.044  (0.925,1.180) | **0.515**  **(0.293,0.903)** | 1.070  (0.942,1.215) | 0.978  (0.861,1.111) | 0.915  (0.799,1.046) | 0.950  (0.795,1.137) | 0.873  (0.707,1.080) |
| **Philippines** | No | 1.050  (0.908,1.213) | 1.026  (0.373,2.824) | **1.477**  **(1.273,1.714)** | **0.693**  **(0.596,0.807)** | **0.646**  **(0.555,0.752)** | 1.009  (0.811,1.256) | **0.698**  **(0.540,0.902)** |
|  | Yes | 1.013  (0.879,1.167) | 1.212  (0.443,3.313) | **1.529**  **(1.322,1.769)** | **0.728**  **(0.628,0.844)** | **0.643**  **(0.554,0.746)** | 1.010  (0.814,1.253) | **0.694**  **(0.538,0.896)** |
| **United Kingdom** | No | 1.005  (0.935,1.081) | 0.917  (0.633,1.328) | **0.911**  **(0.844,0.984)** | **1.080**  **(1.001,1,167)** | **1.130**  **(1.042,1.226)** | 0.986  (0.892,1.091) | **0.880**  **(0.783,0.991)** |
|  | Yes | 0.984  (0.918,1.054) | 0.904  (0.652,1.254) | **0.915**  **(0.850,0.984)** | **1.078**  **(0.628,0.844)** | **1.102**  **(1.021,1.190)** | 0.961  (0.873,1.056) | **0.896**  **(0.803,0.999)** |
| **Vietnam** | No | 0.862  (0.717,1.036) | 1.327  (0.315,5.593) | **1.542**  **(1.278,1.859)** | **0.643**  **(0.532,0.778)** | **0.646**  **(0.534,0.781)** | 0.903  (0.695,1.174) | **0.469**  **(0.282,0.780)** |
|  | Yes | 0.854  (0.711,1.025) | 1.521  (0.364,6.349) | **1.606**  **(1.333,1.936)** | **0.686**  **(0.568,0.829)** | **0.638**  **(0.528,0.771)** | 0.872  (0.672,1.132) | **0.474**  **(0.286,0.785)** |
| **Other mainly English-speaking countries** | No | 1.079  (0.939,1.239) | 0.827  (0.370,1.851) | 1.130  (0.978,1.305) | 0.893  (0.772,1.033) | 0.948  (0.812,1.107) | 0.845  (0.696,1.027) | 1.013  (0.810,1.268) |
|  | Yes | 1.061  (0.928, 1.213) | 0.882  (0.418,1.858) | **1.157**  **(1.007,1.330)** | 0.907  (0.789,1.043) | 0.941  (0.811,1.092) | 0.836  (0.694,1.008) | 1.035  (0.841,1.275) |
| **Other mainly non-English speaking countries** | No | **1.123**  **(1.064, 1.184)** | **0.491**  **(0.385,0.627)** | 0.958  (0.906,1.014) | 0.984  (0.930,1.041) | 0.953  (0.899,1.011) | **0.801**  **(0.745,0.863)** | **0.787**  **(0.720,0.859)** |
|  | Yes | **1.111**  **(1.055, 1.171)** | **0.554**  **(0.440,0.697)** | 0.983  (0.930,1.038) | 1.028  (0.974,1.086) | 0.952  (0.899,1.009) | **0.789**  **(0.734,0.847)** | **0.808**  **(0.742,0.880)** |

* Adjusted for age, SES, diagnostic year, comorbidity, and stage (for treatment outcomes): Multivariate logistic regression, excluding multiple primary cancers (see text).

** Adjusted for age, SES, diagnostic year, stage, comorbidity, and first round treatment by surgery, radiotherapy and systemic therapy: Multivariate proportional hazards regression, excluding multiple primary cancers. Date of censoring of live cases 5 years post-diagnosis or on April 30^th^ 2020, whichever came first (see text).

CIs – confidence intervals.
